# The novel use of a vacuum-assisted closure dressing in the management of Fournier's gangrene

**DOI:** 10.1308/rcsann.2023.0102

**Published:** 2024-11-01

**Authors:** L Condell, N Doolan, M McMonagle

**Affiliations:** ^1^ St Vincent’s University Hospital, Dublin, Ireland; ^2^ Beaumont Hospital, Dublin, Ireland; ^3^ University Hospital Waterford, Ireland; ^4^ Imperial College Healthcare NHS Trust, UK

## BACKGROUND

**Figure 1:**
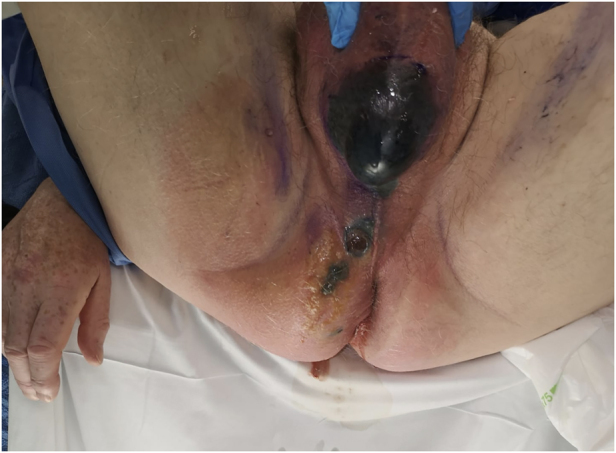
Initial presentation of Fournier's gangrene of the scrotum and perineum

Fournier's gangrene is an aggressive condition caused by polymicrobial infection, in which infected gangrene spreads rapidly along the fascial planes of the perineum and genitalia (Figure 1).^1,2^ It requires immediate and radical debridement of all necrotic tissue with concurrent broad-spectrum antibiotic cover.^2,3^ Frequent relook procedures involving further debridement are often required, resulting in an extensive wound that can be challenging to manage.^4,5^

## TECHNIQUE

**Figure 2:**
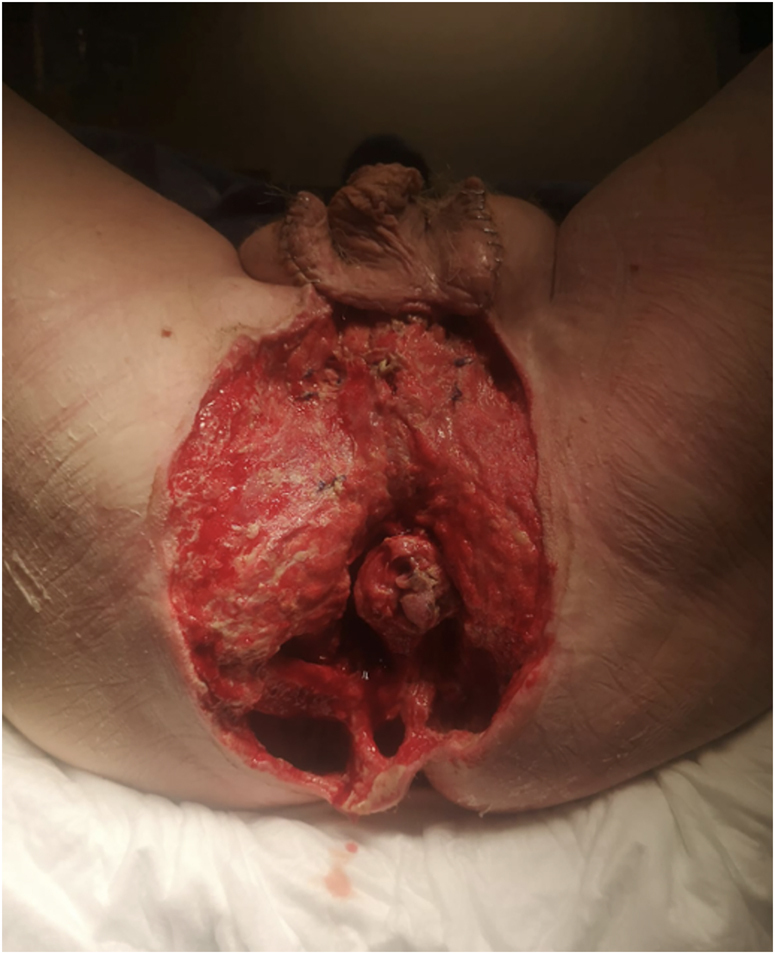
Debridement of necrotic tissue back to viable tissue (note testicles temporarily relocated into inguinal pouch)

**Figure 3:**
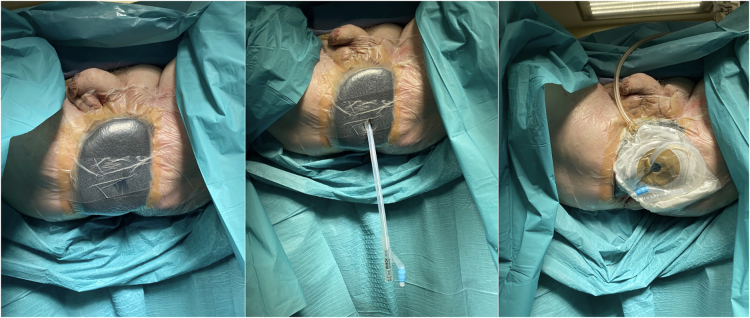
Placement of black vacuum-assisted closure foam over debrided area with insertion of a Foley catheter into the anal canal and overlying stoma bag in situ

All infected, necrotic tissue is debrided until viable tissue is reached (Figure 2). A vacuum-assisted closure (VAC) sponge is placed over the wound and secured at the wound edge with staples. A window is cut in the sponge to accommodate the anal opening. Similarly, a coordinating window is cut in the overlying plastic adhesive. A Foley catheter is placed into the anal canal. A stoma bag is placed over the anal opening (with the other end of the Foley catheter placed inside the bag). The drainage tubing is placed and connected to the canister (Figure 3).

## DISCUSSION

Negative-pressure wound therapy with faecal diversion is commonly used in the management of Fournier's gangrene.^5^ The technical details that distinguish this technique from other methods of management are: (i) placement of a Foley catheter into the anal canal; and (ii) placement of a stoma bag over the protruding end of the Foley catheter. This acts as a vent, maintaining the VAC seal while enabling the continuous collection of secretions from the anal canal. This reduces congestion of the sponge with secretions while maintaining negative pressure, which aids in clean and effective wound healing, tissue granulation and neovascularisation.
